# Cell-Specific DNA Methylation Markers in Plasma cfDNA Reveal Diagnostic Potential for Head and Neck Cancer

**DOI:** 10.7150/jca.133470

**Published:** 2026-07-13

**Authors:** Tianci Wang, Fei Han, Yuxuan She, Mengju He, Yicen Ding, Qi Mei, Yani Kang

**Affiliations:** 1 School of Biomedical Engineering, Bio-ID Center, Shanghai Jiao Tong University, Shanghai, 200240, China.; 2 Shanxi Province Cancer Hospital/Shanxi Hospital Affiliated to Cancer Hospital, Chinese Academy of Medical Sciences/Cancer Hospital Affiliated to Shanxi Medical University, Taiyuan,030013, China.; 3 Shanghai Starriver Bilingual School, Shanghai, 201108, China.; 4 Cancer Center, Shanxi Bethune Hospital, Shanxi Academy of Medical Sciences, Tongji Shanxi Hospital, Third Hospital of Shanxi Medical University, Taiyuan, 030032, China.; 5 Department of Oncology, Tongji Hospital, Tongji Medical College, Huazhong University of Science and Technology, Wuhan, 430030, China.

**Keywords:** head and neck cancer, cfDNA, DNA methylation, liquid biopsy

## Abstract

**Purpose:**

Head and neck cancer ranks as the seventh most common type of cancer worldwide. For patients diagnosed at advanced stages, the five-year survival rate remains below 40%, underscoring the urgent need for early detection strategies. However, current diagnostic approaches face considerable limitations. Furthermore, tissue biopsy remains invasive and impractical for routine screening. In light of these challenges, this study aims to identify and validate plasma cell-free DNA (cfDNA) methylation markers capable of enabling auxiliary assessment and facilitating prognosis assessment in head and neck cancer.

**Methods:**

To achieve this goal, we first analyzed whole-genome bisulfite sequencing (WGBS) data from 205 samples covering 39 healthy tissue types. This comprehensive analysis was conducted to establish a baseline methylation landscape and identify methylation sites specifically associated with head and neck cells. Subsequently, differentially methylated CpG sites were screened by comparing head and neck tissues with other healthy tissues. To enhance the robustness and specificity of potential markers, more than 5 adjacent cell-specific methylated CpG sites located within 150 base pairs of each other were clustered into composite methylation markers, thereby reducing background noise and improving signal detection. These candidate markers then underwent a stringent specificity test to confirm their exclusive enrichment in head and neck tissues. Finally, one of the most promising markers was experimentally validated in plasma cfDNA samples collected from head and neck cancer patients compared with healthy controls, using targeted bisulfite sequencing to assess their detection performance in liquid biopsies.

**Results:**

Through comprehensive analysis of WGBS data from healthy tissue samples, a total of 1,157 tissue-specific methylation sites were identified, including 146 hypermethylated and 1,011 hypomethylated sites exclusive to head and neck tissues. By clustering adjacent CpG sites within a 150-base-pair window, these were consolidated into 11 composite methylation markers, each representing a distinct genomic region with coordinated methylation patterns. Subsequent specificity testing confirmed that all composite markers exhibited strong cell-type specificity, with minimal background methylation signals detected across other tissue types. This high specificity underscores their potential for accurate tissue-of-origin inference. Validation experiments in plasma cfDNA samples further demonstrated that methylation fragments corresponding to these composite markers were consistently detectable in cancer patients but entirely absent in healthy individuals.

**Conclusion:**

This study systematically characterizes cell-specific methylation signatures of head and neck tissues and provides preliminary evidence for the feasibility of utilizing composite cfDNA methylation markers for non-invasive cancer detection. By integrating multiple adjacent CpG sites into composite markers, our approach achieves enhanced specificity and robustness compared to conventional single-site markers, providing a strategy to overcome limitations associated with tumor heterogeneity and low cfDNA abundance in early-stage disease. These findings not only provide a methodological framework for cfDNA-based auxiliary assessment but also offer promising candidate biomarkers for tracing the tissue of origin in head and neck cancer. This work is limited by a small sample size and pending further investigation in larger, prospective cohorts. However, this work illustrates the potential of cell-specific methylation analysis to contribute to improved disease monitoring and tissue-of-origin determination.

## Introduction

Head and neck cancer (HNC) is the seventh most common type of cancer worldwide, causing approximately 500,000 deaths annually. The five-year survival rate for advanced-stage HNC is less than 40% 1. HNC originates from malignant tumors in the head and neck regions, primarily arising from the oropharynx (10.64%), nasopharynx (8.33%), larynx (4.82%), and thyroid (76.21%) 2. The predominant subtype of HNC is head and neck squamous cell carcinoma (HNSCC), accounting for over 90% of HNC cases. Other common types include mucosal melanoma and nasopharyngeal carcinoma 1, 3.

Currently, HNC diagnosis primarily relies on imaging-based assessments and histopathological confirmation through tissue biopsy. However, imaging-based assessment lacks sufficient sensitivity for early-stage detection, and many patients are already at intermediate or advanced stages when diagnosed through tissue biopsy 4, 5. Tumor markers used in HNC diagnosis include squamous cell carcinoma antigen (SCCA), carcinoembryonic antigen (CEA), and human papillomavirus DNA (HPV-DNA). However, these conventional biomarkers suffer from limited specificity, poor sensitivity in early-stage disease, and an inability to reflect tumor heterogeneity or localize the tumor origin without complementary imaging techniques 6. Strategies for detecting circulating HPV-DNA have shown strong performance for diagnosis and post-treatment surveillance in HPV-driven cancers and are currently among the most mature blood-based biomarkers in the HNC field. However, they are inherently restricted to HPV-positive disease and do not address the broader biological heterogeneity of HPV-negative HNC 7, 8. Therefore, finding novel noninvasive and specific biomarkers for the early detection and prognostic evaluation of HNC is an urgent need.

Liquid biopsy refers to the collection and analysis of various body fluids, such as blood, saliva, urine, pleural effusion, and cerebrospinal fluid 9. It enables the detection of multiple biomarkers, including circulating tumor cells (CTCs), extracellular nucleic acids such as cell-free DNA (cfDNA), extracellular vesicles (EVs), tumor-educated platelets (TEPs), metabolites, proteins, and antigens 10. Compared with conventional tissue biopsies, liquid biopsies are less invasive, more convenient, cost-effective, and allow for repeated sampling, making them useful for early cancer screening and monitoring treatment response 11.

cfDNA, which was first discovered in human blood by Mandel and Metais in 1948 12, is highly fragmented double-stranded DNA with a length of 166bp as peak 13, 14. cfDNA is released into circulation through apoptosis, necrosis, or active secretion, carrying genetic and epigenetic information from the source cells 13-15. It is a real-time biomarker of physiological status with a 16-150 minutes half-life in circulation 16. In healthy individuals, a CpG site in the same genome location may display different methylation levels in the DNA from different tissues or different types of cells 17. These unique methylation signatures are highly stable and are often maintained even in diseased cells 18, making them valuable for inferring the tissue origin of cfDNA. Many methods for tracing cfDNA origination have been developed and cfDNA has been applied in the research of many other cancers and diseases 15, 19-29. Studies have shown that cfDNA in healthy individuals primarily originates from erythrocyte progenitors, granulocytes, monocytes/macrophages, endothelium, hepatocytes and lymphocytes. When HNC occurs, head and neck tissues will be affected, tumor and paracancerous cells will die and release DNA, thus the tissue contribution to cfDNA will shift. The proportion derived from the affected head and neck tissues will increase. As a result, we can use these biomarkers of healthy tissues to indicate tissue damage and the occurrence of cancer 19-21.

Existing liquid-biopsy approaches for HNC largely fall into several groups. Multiple studies have evaluated tumor-associated methylation panels. Most of them focused on the abnormal methylation patterns in circulating tumor DNA (ctDNA) 30-33. Though ctDNA increases as the tumor progresses, ctDNA usually accounts for less than 0.1% of total cfDNA 11. The key challenge to be faced is the identification of ctDNA in extremely small amounts, and is affecting the advancement of downstream analysis and clinical application 34. Besides, there are now commercial or near-clinical cfDNA methylation platforms, although most are not HNC-specific. Klein et al. use targeted cfDNA methylation profiling combined with machine learning to detect a cancer signal and predict cancer signal origin, but more data is needed to overcome the problem of being identified to multiple origins 35. Rather than using tumor-associated methylation patterns, cell-specific markers use data from more accessible normal tissues. Besides, while tumor DNA has higher variability, some cell-specific methylation signatures are highly stable and are often maintained even in diseased cells and can offer stable cell-of-origin resolution 18. The complementarity of the two strategies makes cell-specific marker an important supplement to the existing cancer detection.

**Figure 1:**
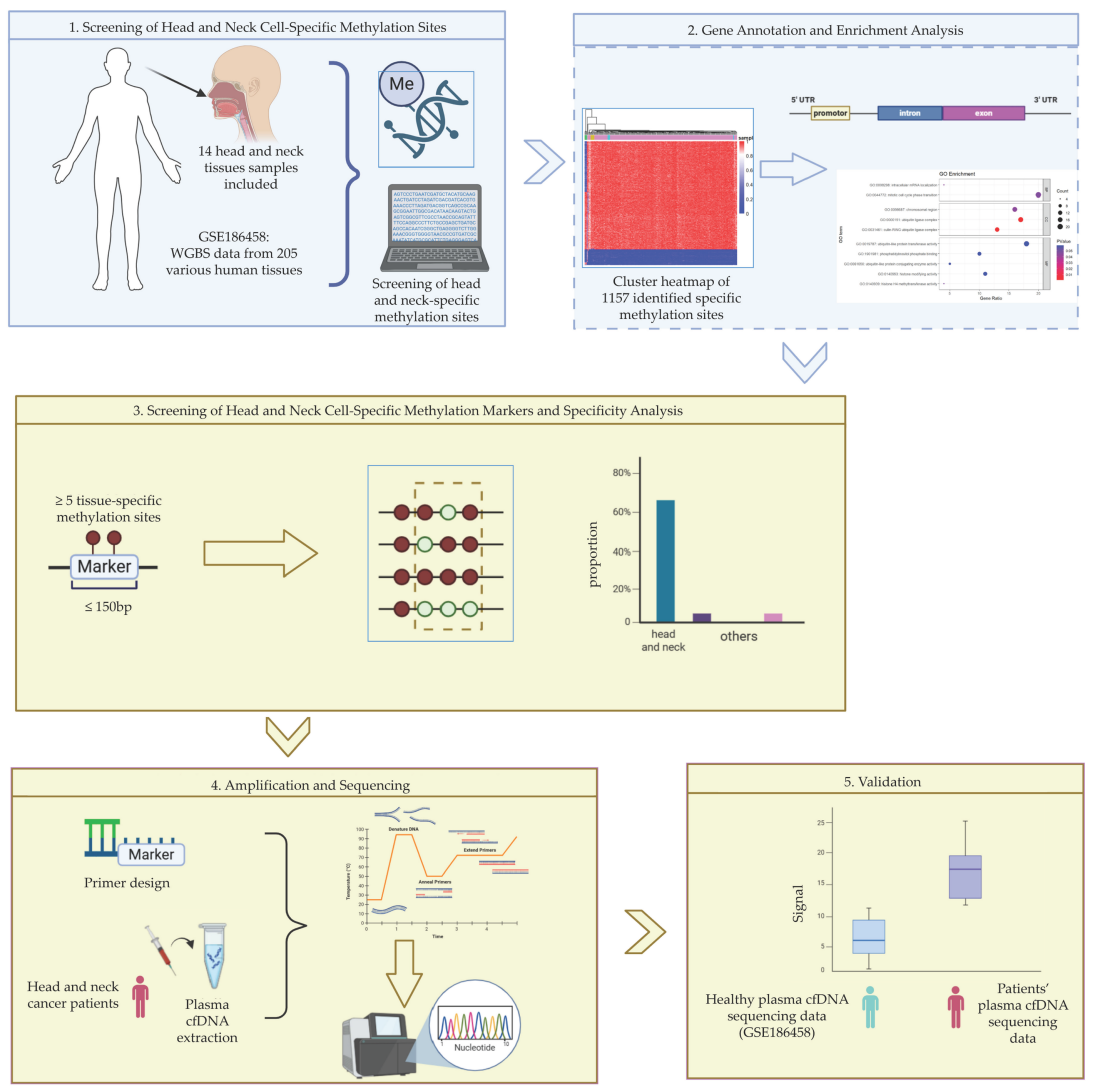
Workflow of experiment and analysis.

Herein, we identified cell-specific methylation sites of cells from head and neck tissues using available cfDNA methylation sequencing data from different tissues of healthy individuals (GSE186458). Based on these sites, we further screened for cell-specific methylation biomarkers composed of more than 5 cell-specific CpG sites within 150 bp, and analyzed their cell type specificity. To validate the performance of the identified biomarkers, plasma cfDNA samples from HNC patients were collected and target regions of the candidate biomarkers were amplified and sequenced. The comparison between sequencing results from patients and from healthy individuals showed the specificity and diagnostic potential of the identified markers. The overall workflow of the study is illustrated in Figure 1.

## Methods

### Public Data Collection

We collected methylation sequencing data from various tissues and cell types available in the GEO database (GSE186458) 19. This dataset includes whole-genome bisulfite sequencing (WGBS) data from 205 samples representing 39 distinct tissues or cell types. Among them, 14 samples were related to head and neck, including three thyroid epithelial samples, one pharyngeal epithelial sample, one laryngeal epithelial sample, five tonsillar epithelial samples, and four lingual epithelial samples.

An independent database of WGBS data from different carcinomas including thyroid carcinoma (GSE212391) 36 was collected to evaluate the performance and stability of thyroid markers in tumor cells.

This study also used WGBS data from 23 independent healthy individuals from the GEO database (GSE186458) 19 to compare with collected patient cfDNA.

### Screening and Analysis of Head and Neck Cell-Specific Methylation Sites

Based on the collected dataset, we utilized wgbstools (v0.2.2) to generate a matrix of methylation levels. We utilized Python (v3.6.2) to filter CpG sites with the following criterion: High methylation specificity, defined as methylation levels > 0.8 in all target samples and methylation levels < 0.5 in background samples; Low methylation specificity, defined as methylation levels < 0.5 in all target samples and methylation levels > 0.8 in background samples.

Then we utilized R (v4.5.2) to conduct gene annotation, CpG island annotation, Gene Ontology (GO) and Kyoto Encyclopedia of Genes and Genomes (KEGG) enrichment analyses, Human Phenotype Ontology (HPO) annotation and visualization on candidate CpG sites. Details of the pipeline are provided in Supplementary Materials.

### Identification and Analysis of Head and Neck Cell-Specific Methylation Biomarkers

Based on the previously identified CpG sites with cell specificity, we further filtered regions containing five or more specific CpG sites within a 150 bp window using Python. Filtered regions were defined as cell-specific markers.

Once candidate marker regions were identified, we assessed their specificity using the collected data of 39 tissues or cell types. We extracted sequencing reads that cover all CpG sites of each candidate marker. For each sample, we calculated the proportion of reads that showed fully methylated or fully unmethylated patterns across all marker CpG sites using Python. An independent database (GSE212391) was utilized to evaluate the performance of thyroid markers. We utilize R and Microsoft Excel to visualize the performance of each marker. These evaluations allowed us to select the most specific and informative methylation biomarkers for downstream validation.

### Primer Design and PCR Optimization

For the selected specific methylation biomarkers, the corresponding gene sequences were retrieved from the UCSC Genome Browser (https://genome.ucsc.edu/index.html). Each biomarker fragment, along with approximately 200 bp upstream and downstream flanking regions, was used as input for primer design using the MethPrimer tool (https://www.methprimer.com/) to generate PCR primers suitable for bisulfite-converted DNA. The designed primers were subsequently validated using Primer3Plus (https://www.primer3plus.com/index.html). During primer design for targeted bisulfite sequencing (TBS), it's recommended to avoid primers covering CpG sites. In addition, the PCR product size was chosen to be close to the length of cfDNA fragments. However, for certain biomarker regions where dense CpG sites in the flanking sequences made it impossible to design suitable primers that completely avoid CpG sites, we adopted primers covering one CpG site as an alternative strategy. Specifically, we designed two sets of primers targeting the methylated and unmethylated versions in consideration of different methylation status. The final primer sequences were synthesized by Shanghai Sangon Biotech Co., Ltd.

For each primer, we performed a pre-experiment to confirm the specificity and the optimum amplification temperature. For primers with poor performance, redesign or adjustment of primer concentration was performed. Based on the results of gel electrophoresis, primers with high specificity were selected, and the optimal amplification conditions were determined. These conditions are summarized in Table S1.

### Collection of Plasma cfDNA from HNC Patients

Plasma samples from HNC patients (n=3) were obtained from Shanxi Province Cancer Hospital. All patients had signed informed consent. With the patient's consent, we supplement partially desensitized information in Table S5. This study was approved by the ethics committee of Shanxi Province Cancer Hospital (Ethical Approval Number JC2024022). After isolation from patients, all samples were stored at -80 °C until cfDNA extraction. According to the manufacturer's instructions, cfDNA was extracted using the QIAamp® Circulating Nucleic Acid Kit (Qiagen, Germany) and the DNA concentration was quantified using the Qubit® dsDNA HS Kit (Thermo Fisher, USA). Extracted cfDNA was stored at -80 °C for subsequent analysis.

### Construction of Methylation Sequencing Libraries

To construct the methylation sequencing libraries, firstly we performed bisulfite conversion, followed by amplification of the marker region, and then we performed library construction, and finally the purified libraries were sent to conduct quality control and sequencing. In detail, bisulfite conversion of plasma cfDNA was performed using the EZ DNA Methylation-Gold™ Kit (Zymo Research, USA), amplification of the marker region was performed using the TaKaRa™ Premix Hot Start Ex Taq (Takara Bio, Japan), and purification was performed using Backman™ Ampure XP Beads (Beckman Coulter, USA), following the manufacturer's instructions.

Amplified cfDNA samples were then used to construct the methylation sequencing libraries, using NEBNext® Ultra II Kit (New England Biolabs, USA). Necessary purification was performed using Backman™ Ampure XP Beads and DNA concentration quantification was performed using the Qubit® dsDNA HS Kit (Thermo Fisher, USA). Quality assessment was performed using Agilent 2100 (Agilent, USA), and sequencing was conducted on the NovaSeq 6000 platform (Illumina, USA) with paired-end 2×150 bp reads.

### Analysis of Sequencing Data

The sequencing data of healthy individuals was provided in the form of PAT files aligned to the human genome (hg38). We take the same pipeline to generate PAT files from our sequencing raw data. Detailed pipeline is in Supplement Material files.

Using the same approach as validating biomarkers' specificity across 205 samples, we analyzed both the healthy plasma cfDNA samples and the patient cfDNA samples. We calculate the fraction of marker reads relative to total mapped reads, and examined the presence of biomarker fragments in the cfDNA of HNC patients compared to healthy individuals. Theoretically, healthy individuals should not carry these biomarker fragments, while in patients, the increased cell death in head and neck tissues would lead to the release of DNA into the plasma, thus making the biomarker fragments detectable in patient's samples.

The number of cfDNA that originates from head and neck tissues per ml of plasma in a sample was calculated by the following formula: N_tissue_ = R_marker_ * C_cfDNA_ / (L_cfDNA_ * M_bp_). *N_tissue_* is the number of cfDNA that originates from head and neck tissues (copies/µL), *R_marker_* is the fraction of marker reads relative to total mapped reads in the sample, *C_cfDNA_* is the concentration of cfDNA in plasma (ng/µL), *L_cfDNA_* is assumed to be the peak length of cfDNA (bp) 13, 14 and *M_bp_* is taken as 650 Da/bp, the mean weight of double-strand DNA 37 and is equal to 1.08e-12 ng/bp.

## Results

### Analysis of Cell-Specific Methylation Sites of Head and Neck tissues

The data we collected contains WGBS data from 205 samples representing 39 different tissues or cell types. Among them, 14 samples are related to head and neck tissues, including 3 thyroid epithelial samples, 1 laryngeal epithelial sample, 1 pharyngeal epithelial sample, 5 tonsillar epithelial samples, and 4 lingual epithelial samples.

**Figure 2:**
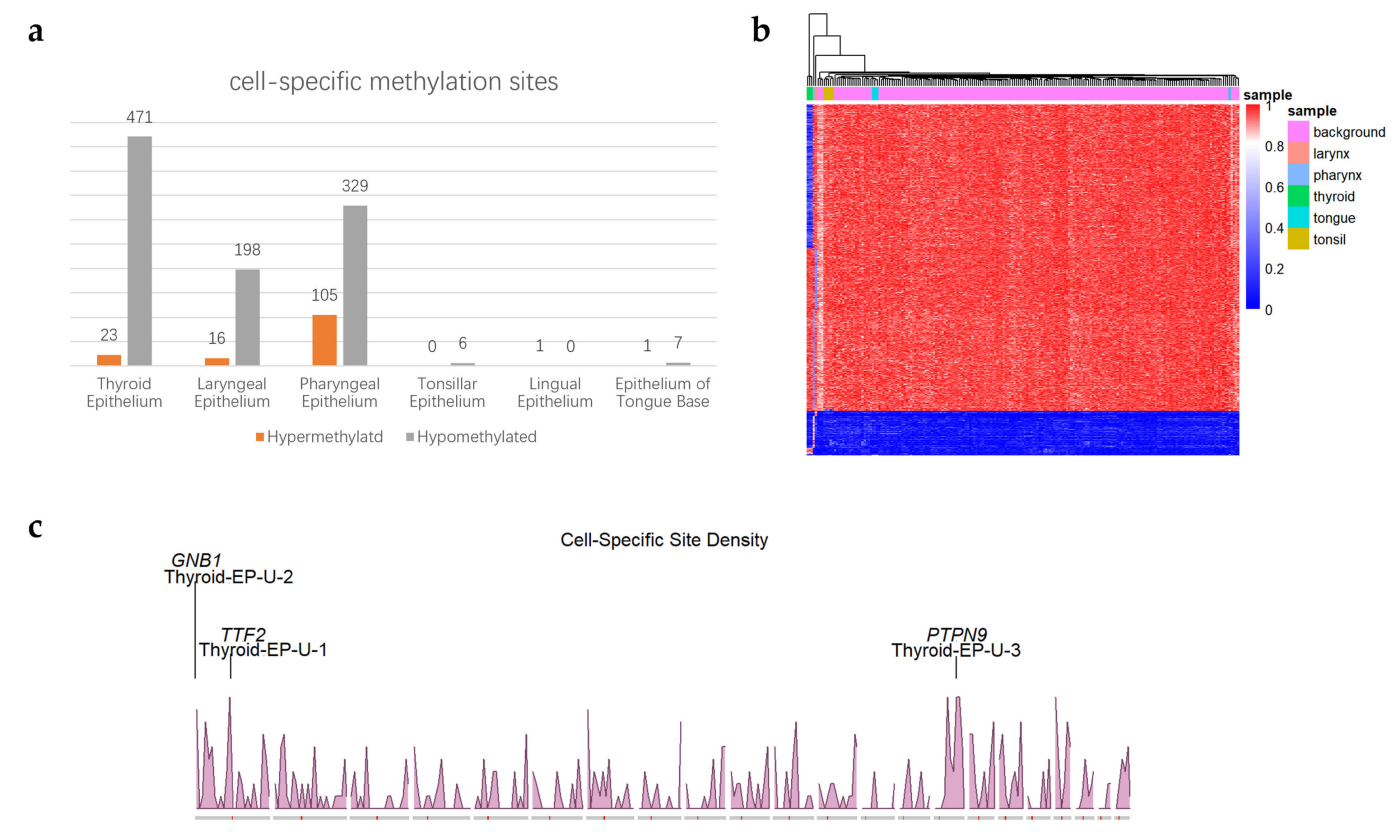
Overview of identified cell-specific methylation sites. (a) Count of identified cell-specific methylation sites. The x-axis represents different head and neck cell types. The y-axis represents the number of identified cell-specific methylation sites; (b) Hierarchical clustering heatmap of specific sites in 205 cell type samples. The x-axis represents different cell types, with colors distinguishing each cell type. The y-axis represents the screened cell type-specific methylation sites, where each row corresponds to the methylation level of a site across different cell types. The color gradient from blue to red indicates methylation levels from low to high; (c) Density of identified cell-specific hypomethylated sites of thyroid epithelium. The x-axis represents 22 chromosomes (chromosome X and chromosome Y are excluded). The y-axis represents the density of identified cell-specific sites with a window of 10e6 bp. 3 identified markers are located at the chromosomes with names and associated genes.

Based on the WGBS data from the various tissues and cell types, a total of 1,157 cell type-specific CpG sites were identified, including 146 hypermethylated specific sites and 1,011 hypomethylated specific sites. As summarized in Figure 2a, thyroid epithelium had the most cell-specific methylation sites. A hierarchical clustering heatmap based on these sites is shown in Figure 2b, which demonstrates that thyroid epithelium, laryngeal epithelium, and pharyngeal epithelium are distinguishable from other tissues in the clustering results.

The results showed that certain sample types, such as tonsillar epithelium and lingual epithelium contained relatively few cell-specific methylation sites. According to previous study 19 and based on our own clustering analysis of CpG methylation levels across 205 tissue samples, it was observed that some head and neck epithelial cells share similar methylation patterns with upper digestive tract epithelial cells, such as esophageal epithelial cells. As a result, it was difficult to independently identify unique methylation markers for these particular tissue types.

**Figure 3:**
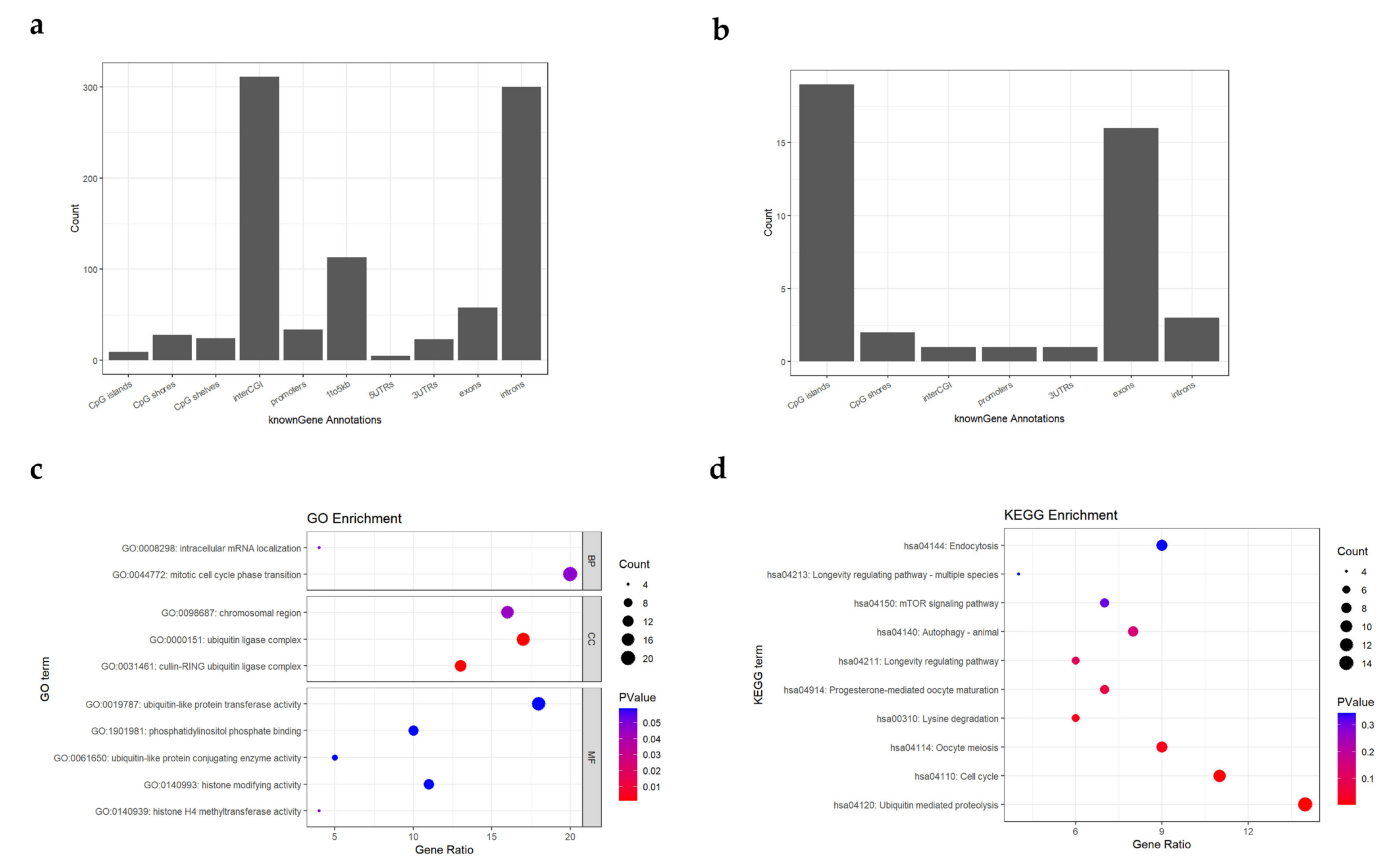
Result of gene annotations and enrichment analysis. (a-b) Distribution of gene annotations for thyroid epithelial cell-specific hypomethylated (a) and hypermethylated (b) sites. The x-axis represents the genomic annotation categories: For CpG island-related annotations, the categories include: CpG islands (CGIs), CpG shores (regions within ±2 kb of CGIs), CpG shelves (regions 2-4 kb upstream or downstream of CGIs), and inter-CGI regions (regions outside the above categories); For gene-related annotations, the categories include: the 1-5 kb upstream region of the gene (1to5kb), promoters (within 1 kb upstream of the transcription start site), 5′ untranslated regions (5′UTRs), 3′ untranslated regions (3′UTRs), exons, and introns. The y-axis indicates the number of specific methylation sites annotated to each genomic region; (c-d) GO enrichment analysis (c) and KEGG enrichment analysis (d) of hypomethylated cell-specific sites in thyroid epithelial cells. Dot size represents the number of enriched genes; Dot color reflects the significance level (p-value) of enrichment; The x-axis indicates the proportion of enriched genes relative to the total number of genes in each term; The y-axis lists the names of the enriched GO or KEGG terms.

Density of identified cell-specific methylation sites was shown in Figure 2c and Figure S1. Through the density map, we found a large number of clustered specific sites on chromosomes 1 and 15. The identified CpG sites were annotated based on genomic features, and bar plots were generated for cell types with more than 15 specific methylation sites, as shown in Figure 3a-b and Figure S2. The results revealed that hypomethylated specific sites were primarily located in intronic and non-CpG island regions, and showed similar distribution patterns across different tissue types. In contrast, the distribution of hypermethylated specific sites varied between tissue types: In thyroid epithelial samples, the sites were predominantly enriched in exonic regions and CpG islands; In pharyngeal epithelium, they were mainly found in intronic regions and CpG islands; In laryngeal epithelium, the sites were concentrated in intronic and inter-CGI regions.

GO and KEGG enrichment analyses were performed on the identified cell-specific methylation sites. For visualization, the top 10 enriched terms with the lowest q-values were plotted as shown in Figure 3c-d and Figure S3. From the visualizations, we observed that in the GO analysis of hypomethylated specific sites in thyroid epithelial cells. The most significantly enriched GO terms were: Cullin-RING Ubiquitin Ligase Complex and Ubiquitin Ligase Complex. In the KEGG analysis, the most significant pathway was Ubiquitin-Mediated Proteolysis. These results suggest that genes associated with hypomethylated specific sites in thyroid epithelial cells may be related to their protein secretion functions. For laryngeal and pharyngeal epithelial cells, the two most significantly enriched Cellular Component (CC) terms in the analysis of hypomethylated specific sites were Chromosomal Region, Chromosome and Centromeric Region. These same terms were also prominently enriched in the thyroid epithelial cell analysis, indicating that the genes corresponding to these hypomethylated sites may be functionally associated with chromosomal structure and organization. In the GO analysis of hypermethylated specific sites in pharyngeal epithelial cells, the most enriched terms were Spinal Cord Development and Forebrain Development. These terms are associated with development and differentiation, suggesting that genes in these regions may be transcriptionally silenced by hypermethylation in highly differentiated cells. Thus, these hypermethylated specific sites may represent cell-specific silencing regions in pharyngeal epithelial cells.

From the HPO annotation, cell-specific sites related gene were significant enriched in head and neck diseases, including Thyroid Hypoplasia, Congenital Hypothyroidism, Laryngeal Carcinoma and so on (Table S2). It is indicated that these CpG sites may play an important role in the development and functional patterns of the head and neck.

### Identification of Cell-Specific Methylation Biomarkers of Head and Neck tissues

**Figure 4:**
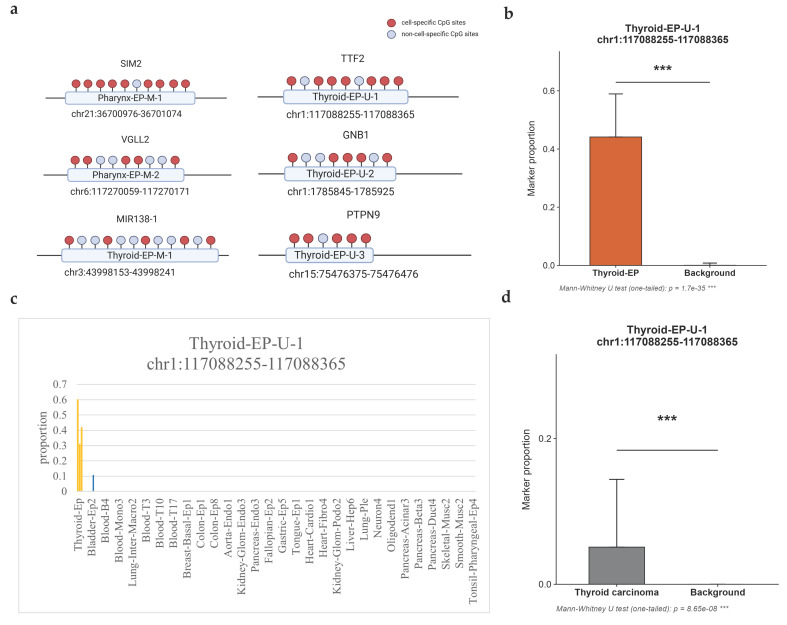
Overview of identified markers and specificity analysis. (a) Composition of each marker; (b) Proportional distribution of Thyroid-EP-U-1 biomarkers in thyroid epithelium compared with other samples, the left bars represent the target cell samples, while the right bars represent background samples; (c) Proportional distribution of Thyroid-EP-U-1 biomarkers across 205 samples, left orange bars represent target cell samples, while other blue bars represent other background samples; (d) Proportional distribution of Thyroid-EP-U-1 biomarkers in thyroid carcinoma compared with other carcinoma, the left bars represent the target samples, while the right bars represent background samples; In (b) to (d), The y-axis indicates the proportion of biomarker reads among all reads covering the target sites; In (b) and (d), the error bars indicates the within-group standard deviation, the p-value is calculated by Mann-Whitney U test, and the *** indicates that p-value is lower than 0.001.

By further selecting the identified cell-specific methylation sites, regions containing five or more adjacent specific CpG sites within 150 bp were defined as cell-specific biomarkers, resulting in a total of 11 biomarkers, as shown in Table S3. In some genes, there were regions containing multiple identified biomarkers. For example, in chr6: 117270014-117270171, 6 cell-specific CpG sites within 157bp constituted 2 biomarkers, and in chr3: 43998583-43997882, 18 cell-specific CpG sites within 701bp constituted 5 biomarkers. In these regions, only the biomarker that covers the most specific sites or has the shortest length was retained for following analysis, as summarized in Table 1. Each marker covered multiple CpG sites, but only the identified cell-specific CpG sites were considered parts of the marker, as shown in Figure 4a. The chromosomal locations of the identified markers are shown in Figure 2c and Figure S1, together with the density distribution of specific sites. Several other regions with clustered sites could also serve as potential markers under less stringent criteria.

Using multiple adjacent differentially methylated sites as biomarkers provides higher specificity compared with using multiple individual sites separately. This specificity can be validated by calculating the proportion of sequencing reads in which the target sites are either completely methylated or completely unmethylated. Based on the collected PAT files from 205 different tissues or cell types, a specificity analysis was performed for selected biomarkers. The analysis calculated the proportion of reads covering the target sites and all the target sites were fully methylated or unmethylated, as shown in Table S4 and visualized in Figure 4b-c and Figure S4. Each marker has significant performance in distinguishing target tissues.

Compared with single-site markers, markers composed of multiple adjacent specific CpG sites within a defined base-pair range exhibited a higher signal-to-noise ratio and specificity. These markers were rarely observed in sequencing data from non-target tissues and were almost exclusively detected in the target tissue samples. In an outside database of WGBS data from different carcinomas including thyroid carcinoma (GSE212391), all four identified thyroid markers have significant performance in distinguishing tissues from thyroid carcinoma. The result is summarized in Table S13 and visualized in Figure 4d and Figure S5.

Furthermore, these markers were even rarer in those tissues where cfDNA of healthy individuals originated from (erythrocyte progenitors, granulocytes, monocytes/macrophages, endothelium, hepatocytes and lymphocytes). Based on these findings, if the biomarker is detected in plasma cfDNA, it is likely that the patient has sustained damage in certain body regions, and the damaged site(s) are most probably located in the head and neck.

### Validation on cfDNA from HNC patients' plasma

cfDNA samples extracted from 3 patients' blood plasma are listed in Table S5. Each sample yielded 10 μL of bisulfite-converted DNA. The concentrations were measured and summarized in Table S6, where the concentration of Sample 2 after bisulfite conversion was below the detection limit.

Primers and conditions from Table S1 were used for amplification. The concentrations of the PCR products were quantified and recorded in Table S7. The amplification products were further examined by agarose gel electrophoresis (Figure S6). The amplification products produced bands at the expected size (~180 bp), showing the specificity of the amplification. The amplification products were then subjected to library construction.

During the library construction, quantification was performed at four stages: after PCR product purification, before library amplification, after library amplification, and after post-amplification purification, with the results summarized in Table S8. The constructed libraries were further assessed by agarose gel electrophoresis (Figure S6), which revealed the presence of large DNA fragments. To remove these large fragments, an additional bead purification step was conducted, and the final concentrations were measured again and recorded in Table S8. The products were then sent for sequencing after quality inspection.

After quality assessment and adapter trimming, sequencing reads were aligned to the hg38 reference genome using Bismark. The alignment rates of each sample are summarized in Table S8. Although the overall alignment rates were relatively low, which may be due to DNA fragment degradation caused by bisulfite treatment, all alignment rates were above 50%. The aligned BAM files were subsequently sorted using samtools, and then converted into PAT files using wgbstools.

The Pharynx-EP-M-2 marker consisted of five CpG sites: cg10331029, cg10331030, cg10331033, cg10331034, and cg10331037. To further evaluate methylation patterns, a broader region spanning cg10331029-cg10331038 (ten CpG sites) was analyzed across all libraries. The sequencing depth and methylation levels are shown in Table S11 and visualized in Figure S7. The sequencing depth of each site is beyond 5*10e6.The results indicated that the methylation levels at these CpG sites were generally low and similar across samples. Notably, cg10331038, which was located within the primer sequence, exhibited distinct methylation patterns depending on the primers used in libraries amplified with U primers, the methylation level at this site was close to 0 (Figure S7), whereas in libraries amplified with M primers, the methylation level approached 1 (Figure S7).

To evaluate the consistency of methylation measurements between primer types, Wilcoxon tests were performed on the methylation levels of the same sample amplified with different primers. The Z-test p-values for Sample 1 and Sample 3 were 0.32 and 0.77, respectively, indicating no significant differences. Therefore, methylation data from both primer types for the same sample were merged for subsequent analyses.

**Figure 5:**
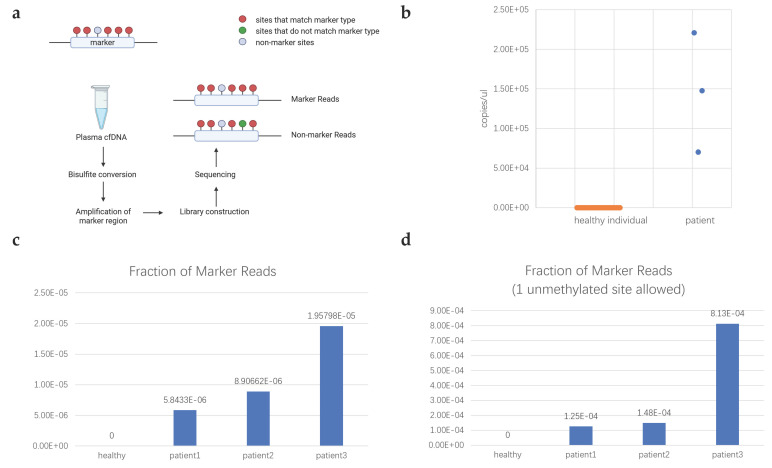
Validation of selected marker. (a) Workflow of validation; (b) Measurement of head and neck cfDNA in plasma of healthy individual and patients with HNC. The x-axis represents the kind of samples. The y-axis represents the measurement of head and neck cfDNA in plasma (copies/μL); (c) Fraction of Pharynx-EP-M-2 marker reads in healthy individuals and patients; (d) Fraction of Pharynx-EP-M-2 marker reads in healthy individuals and patients with 1 unmethylated site allowed. In (c) and (d), x-axis represents the kind of samples. The y-axis represents the fraction of marker reads.

Based on the PAT files, sequencing fragments covering all marker CpG sites were extracted. Then we identified fragments with marker sites fully methylated and at most 1 unmethylated. The counts are summarized in Table S9, showing that marker fragments were detected in all patient samples. The methylation level at each individual site (Table S11) served as a marginal probability to model the expected co-occurrence frequency of the biomarker under the assumption of independence. The observed frequency demonstrated a dramatic enrichment over this random expectation (for each sample, Z-test, *p* < 1×10^-5^). This statistically overwhelming deviation from a stochastic model indicated that the coordinated methylation pattern originates from a specific, non-random biological source. Therefore, the detection of this biomarker in plasma provided evidence for the significant presence of cfDNA derived from a specific pathological tissue origin, which, based on our previous analysis, is most likely to be located in the head and neck region. As shown in Table S11, Mann-Whitney U test was conducted on each single site. Except cg10331029 (p = 0.016), other sites reported no significant (p > 0.1) difference between healthy and patients' cfDNA, indicating single site had no distinguishing ability. By contrast, the same procedure applied to cfDNA sequencing data from 23 healthy individuals revealed no marker fragments (Table S10). Based on the validation dataset, the sensitivity and specificity were calculated as 1, and the ROC analysis is shown in Figure S8. No marker read was detected though 1 unmethylated site was allowed, suggesting that this marker may have potential as a cancer-specific discriminative biomarker. The visualization of the comparison of marker reads fraction between HNC patients and healthy controls was shown in Figure 5c-d. Based on the marker reads fraction, the count of head and neck cfDNA was calculated and shown in Figure 5b. The whole workflow of validation was illustrated in Figure 5a.

## Discussion

In this study, a total of 1,157 cell-specific methylation sites were identified from WGBS data of 205 samples across 39 different tissues, including 146 hypermethylated and 1,011 hypomethylated sites. The results showed that certain sample types, such as tonsillar epithelium and lingual epithelium, contained relatively few cell-specific methylation sites. These epithelial cells might share similar methylation patterns with upper digestive tract epithelial cells, such as esophageal epithelial cells. Shared methylation patterns might lead to false positives. Therefore, no specific markers could be identified for these tissues. These tissues might simultaneously have patterns shared with other HNC tissues and patterns shared with upper digestive tract epithelial cells 19, so overlapping multiple supergroups to identify these tissues might be promising.

The hypomethylated sites were predominantly enriched in intronic regions and non-CpG island regions, exhibiting a conserved distribution across different tissue samples. In contrast, the distribution of hypermethylated sites varied among different tissues.

Introns, as non-coding regions of genes, have traditionally been regarded as important regulators of transcription and RNA splicing. They enhance transcriptional activity by influencing transcriptional rate, nucleotide stability, and mRNA translation efficiency 38. Recent studies have further demonstrated that intronic regions are enriched in numerous regulatory elements (e.g., enhancers, silencers), and their methylation states can significantly affect gene expression. The increase in protein expression mediated by intronic regulatory elements is referred to as intron-mediated enhancement (IME) 39. Hypomethylation is generally associated with an open chromatin state. Therefore, hypomethylation in intronic regions may facilitate the binding of regulatory elements and transcription factors, thereby maintaining cell-specific gene expression patterns.

CpG islands (CGIs) are generally defined as regions longer than 200 bp with a GC content >50% and an observed-to-expected CpG ratio >0.6 40. They are often enriched in promoter regions 41, and DNA methylation in CGIs is commonly associated with cell-specific regulation 42. However, the observation that cell-specific methylation sites were predominantly enriched in non-CpG island regions suggests that methylation variation outside CGIs plays an important regulatory role, potentially reflecting the influence of non-CGI regulatory factors. This enrichment of cell-specific methylation in non-CGI regions is consistent with the observed enrichment in intronic regions. Thus, the cross-tissue conservation of hypomethylated sites may indicate that regulatory elements associated with cell-specific gene expression tend to share similar distribution patterns across different tissues.

A total of 11 markers were obtained by screening for clusters of ≥ 5 specific CpG sites within 150 bp. Related genes include *SIM2*, *VGLL2*,* MIR138-1*,* TTF2*,* GNB1*,* PTPN9*. We explored the Pharynx-EP-M-2 marker, while others were left to be explored in the future. A combination of multiple markers may increase the sensitivity during HNC auxiliary assessment and more information such as the origination and heterogeneity can be offered. In some genes, there are regions containing a large number of cell-specific sites. Though these regions don't meet the filter criterion, they are promising and worth further exploration. The Pharynx-EP-M-2 marker is located at chr6: 117,270,059-117,270,171, comprising 5 CpG sites within the intronic region of *VGLL2*. *VGLL2* is associated with muscle development and is highly active in normal skeletal muscle fibers. Its dysregulated expression has been linked to abnormal cell proliferation and tumorigenesis, and it is frequently implicated in rhabdomyosarcoma 43, 44. The specific methylation of the *VGLL2* intronic region in pharyngeal epithelium may suggest suppression of muscle development.

Using multiple adjacent differentially methylated sites as biomarkers provides higher specificity compared with using multiple individual sites separately. This specificity was validated by calculating the proportion of markers in different cell types. Compared with single-site markers, markers composed of multiple adjacent specific CpG sites within a defined base-pair range exhibited a higher signal-to-noise ratio and specificity. These markers were rarely observed in sequencing data from non-target tissues and were almost exclusively detected in the target tissue samples. Furthermore, these markers were even rarer in those tissues where cfDNA of healthy individuals originated from (erythrocyte progenitors, granulocytes, monocytes/macrophages, endothelium, hepatocytes and lymphocytes). Therefore, if the biomarker is detected in plasma cfDNA, it is likely that the patient has sustained damage in certain body regions, and the damaged site(s) are most probably located in the head and neck. When head and neck tissues are affected by cancer, DNA from these tissues will be released. As a result, the tissue contribution to cfDNA will shift, and the proportion derived from the affected head and neck tissues will increase. Therefore, we can use these biomarkers of healthy tissues to indicate tissue damage and the occurrence of cancer 19-21.

Although the sample size is limited, we can estimate the reasonable thresholds. Studies have shown that cfDNA in healthy individuals primarily (> 1%) originates from erythrocyte progenitors (36%), granulocytes (30%), monocytes/macrophages (20%), endothelium (5%), hepatocytes (3%), lymphocytes (NK cells) (2%) 19-21. The theoretical proportion of marker reads in the healthy cfDNA can be calculated as the sum of each component's proportion in healthy cfDNA multiplied by its corresponding theoretical marker proportion. The detailed calculation and result are summarized in Table S12. Based on the sequencing depth of target region and marker fraction in the sample, we can assess the statistical significance of our judgement. However, most markers are not detected in healthy cfDNA components, so their thresholds are calculated as 0. More data is needed to calculate more precise thresholds.

In the validation of amplification results, fully methylated marker fragments were detected in the Pharynx-EP-M-2 marker region of plasma cfDNA from HNC patients, whereas no fully methylated fragments were detected in the same region of plasma cfDNA from healthy individuals. We used the methylation level at each individual site as a marginal probability to model the expected co-occurrence frequency of the biomarker under the assumption of independence. The observed frequency showed a significant enrichment over the random expectation (for each sample, *p* < 1×10^-5^). The detection of this biomarker in plasma provided evidence for the significant presence of cfDNA derived from a specific pathological tissue origin, which, based on our previous analysis, is most likely to be located in the head and neck region. However, due to the high sequencing depth of cfDNA from patients, no statistically significant clues to support or counter the difference between groups of patients and healthy individuals. Calculated in Table S12, theoretical threshold for Pharynx-EP-M-2 is 0, so we can't calculate the possibility of false negatives for healthy cfDNA. If we use patients' marker fraction as alternative standard, that will be 0.71 according to the hypothesis testing. Deeper sequencing is needed to overcome the problem. This research is a preliminary exploratory investigation and further experiments on other identified markers with a larger validation cohort can be performed.

## Limitations

Limitations still exist. The sample size is small and the difference between existing healthy individuals' cfDNA sequencing depth and patients' is huge. Due to these limitations, there is no statistically significant clue to support or counter the difference between groups of patients and healthy individuals. Further experiments including multiple HNC subtypes and stages are needed to support future clinical applicability.

Future directions can focus on the following aspects. Future studies should include larger cohorts of HNC patients and healthy controls to validate marker sensitivity and specificity. Multi-marker validation and marker panels may further improve diagnostic performance. Combining genomic, transcriptomic, and epigenomic data could deepen insights into the role of methylation markers in tumorigenesis and enhance their diagnostic utility. Improving cfDNA extraction and sequencing workflows, refining primer design strategies, and reducing experimental costs are necessary steps toward clinical translation. Investigating the application of methylation markers in treatment response monitoring and recurrence prediction could provide valuable support for personalized medicine.

## Conclusions

This study focused on the screening and validation of cfDNA methylation diagnostic markers in patients with HNC. Based on WGBS data from the GSE186458 dataset, a total of 1157 cell-specific methylation sites were successfully identified in head and neck tissues, including 146 hypermethylated sites and 1011 hypomethylated sites. Annotation of these sites revealed that cell-specific hypomethylated sites were enriched in non-CpG island regions and intronic regions, showing consistent patterns across different tissues. By applying the criterion of more than 5 adjacent cell-specific methylation sites within 150 bp, a total of 11 methylation marker regions were identified. These markers exhibited strong tissue specificity, being almost exclusively detected in their respective target tissues, thus providing promising molecular markers for the auxiliary assessment of HNC. Through a systematic analysis of cell-specific methylation markers in head and neck cells, combined with experimental validation, this study provides new insights and a methodological framework for the auxiliary assessment of HNCs. While limited by a small sample size and lack of independent validation, this work illustrates the potential of cell-specific methylation analysis to contribute to improved disease monitoring and tissue-of-origin determination, pending further investigation in larger, prospective cohorts.

## Supplementary Material

Supplementary methods, figures and tables.

## Figures and Tables

**Table 1:** Summary of biomarkers for further analysis. The type is denoted as M for hypermethylated biomarkers and U for hypomethylated biomarkers in the Name, while Length and CpGs represent the region length and the number of specific CpG sites contained within that region, respectively.

Name	Length(bp)	CpGs	Chromosomal Region	Associated Gene
Pharynx-EP-M-1	98	10	chr21:36700976-36701074	SIM2
Pharynx-EP-M-2	112	5	chr6:117270059-117270171	VGLL2
Thyroid-EP-M-1	88	5	chr3:43998153-43998241	MIR138-1
Thyroid-EP-U-1	110	7	chr1:117088255-117088365	TTF2
Thyroid-EP-U-2	80	5	chr1:1785845-1785925	GNB1
Thyroid-EP-U-3	101	5	chr15:75476375-75476476	PTPN9

## Data Availability

The data used and analyzed in the current study are deposited at the GEO database (GSE314062). The codes used in the current study are deposited at https://github.com/Ethenone/Cell-Specific-DNA-Methylation-Markers-identification-and-analysis-in-Plasma-cfDNA-from-HNC-patients.

## Abbreviations

CC: cellular component
CEA: carcinoembryonic antigen
cfDNA: cell-free DNA
ctDNA: circulating tumor DNA
CGI: CpG island
CTC: circulating tumor cell
EV: extracellular vesicle
GO: gene ontology
HNC: head and neck cancer
HNSCC: head and neck squamous cell carcinoma
HPO: human phenotype ontology
HPV-DNA: human papillomavirus DNA
KEGG: Kyoto encyclopedia of genes and genomes
SCCA: squamous cell carcinoma antigen
SNR: signal-to-noise ratio
TEP: tumor-educated platelet
WGBS: whole-genome bisulfite sequencing
