# Supplementary material for: Cell-Specific DNA Methylation Markers in Plasma cfDNA Reveal Diagnostic Potential for Head and Neck Cancer
Source: J Cancer. 2026 Jul 13;17(7):1318–30. doi: 10.7150/jca.133470 (PMC13410342; doi:10.7150/jca.133470)
Supplement: Supplementary file 1 — Supplementary methods, figures and tables. [file jcav17p1318s1.zip › Supplementary Material files/detailed method.docx]

Screening and Analysis of Head and Neck Cell-Specific Methylation Sites

Based on the collected dataset GSE186458, we initially performed a preliminary screening of head and neck cell type-specific methylation markers using the find_markers function in wgbstools (v0.2.2). This function was applied to the beta files of all 205 samples, along with a grouping file and a CpG block file. Samples were categorized into “target” (head and neck tissues) and “background” groups. Block file was generate by block function in wgbstools from human genome (hg38). Each block in the CpG block file contained a single CpG site to facilitate site-specific differential analysis. All other parameters were left as default. To further improve marker specificity, we used the beta_to_table function in wgbstools to generate the matrix of methylation level. Then we utilized Python (v3.6.2) to filter selected CpG sites with the following criterion: High methylation specificity, defined as methylation levels > 0.8 in all target samples and methylation levels < 0.5 in background samples; Low methylation specificity, defined as methylation levels < 0.5 in all target samples and methylation levels > 0.8 in background samples.

These candidate CpG sites were visualized with hierarchical clustering heatmaps generated by the pheatmap package (v1.0.12) in R (v4.5.2). Gene annotation and CpG island annotation were performed by the annotatr package (v1.28.0) and the annotate package (v1.80.0), while Gene Ontology (GO) and Kyoto Encyclopedia of Genes and Genomes (KEGG) enrichment analyses were conducted by clusterProfiler package (v4.10.1). Additionally, Human Phenotype Ontology (HPO) annotation was conducted by ChIPseeker package (v1.38.0) to explore the possible association with head and neck diseases.

Analysis of Sequencing Data

The raw output format of sequencing was “fq.gz”. The quality of all three sequencing datasets was assessed using FastQC (v0.11.5) to obtain base quality scores and detect adapter sequences. Next, Trim-Galore (v0.6.10) was used to remove adapters and low-quality reads. After trimming, FastQC was used again to reassess sequence quality. Following adapter removal and quality filtering, Bismark (v0.24.2) was used to align the sequencing reads to the hg38 human reference genome, resulting in BAM files. To determine whether the sequencing data contained the target regions, we used the sort function in samtools (v1.19) to sort the aligned BAM files. After sorting, the bam2pat function in wgbstools was used to convert BAM files into PAT files. We utilize bgzip (v1.9) and tabix (v1.9) to generate zip files and index files to accelerate operation.
