# Supplementary figures and images for: Cell-Specific DNA Methylation Markers in Plasma cfDNA Reveal Diagnostic Potential for Head and Neck Cancer
Source: J Cancer. 2026 Jul 13;17(7):1318–30. doi: 10.7150/jca.133470 (PMC13410342; doi:10.7150/jca.133470)

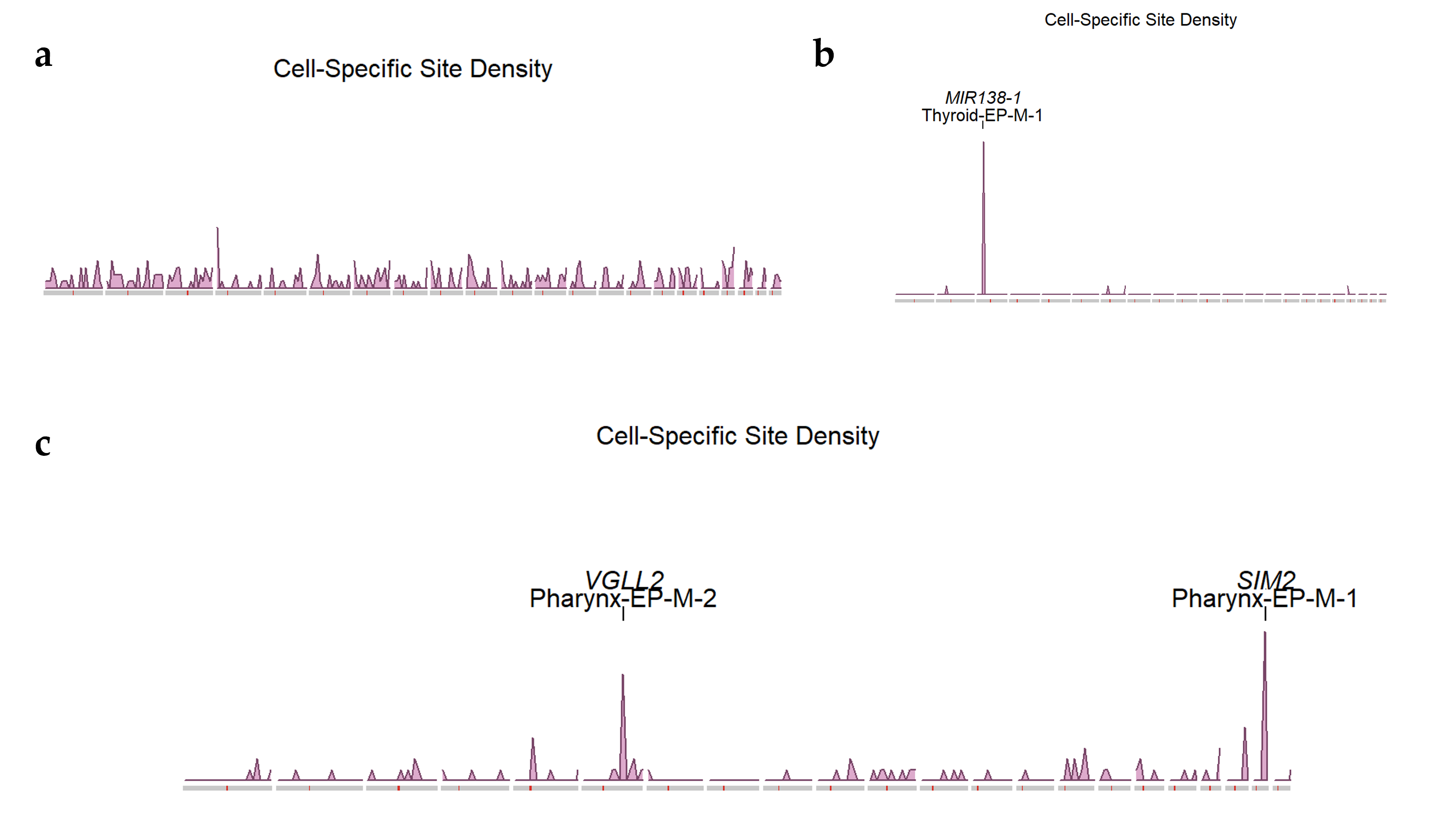

Supplement: Supplementary file 1 — Supplementary methods, figures and tables. [file jcav17p1318s1.zip › Supplementary Material files/Figures/FigureS1.tif]

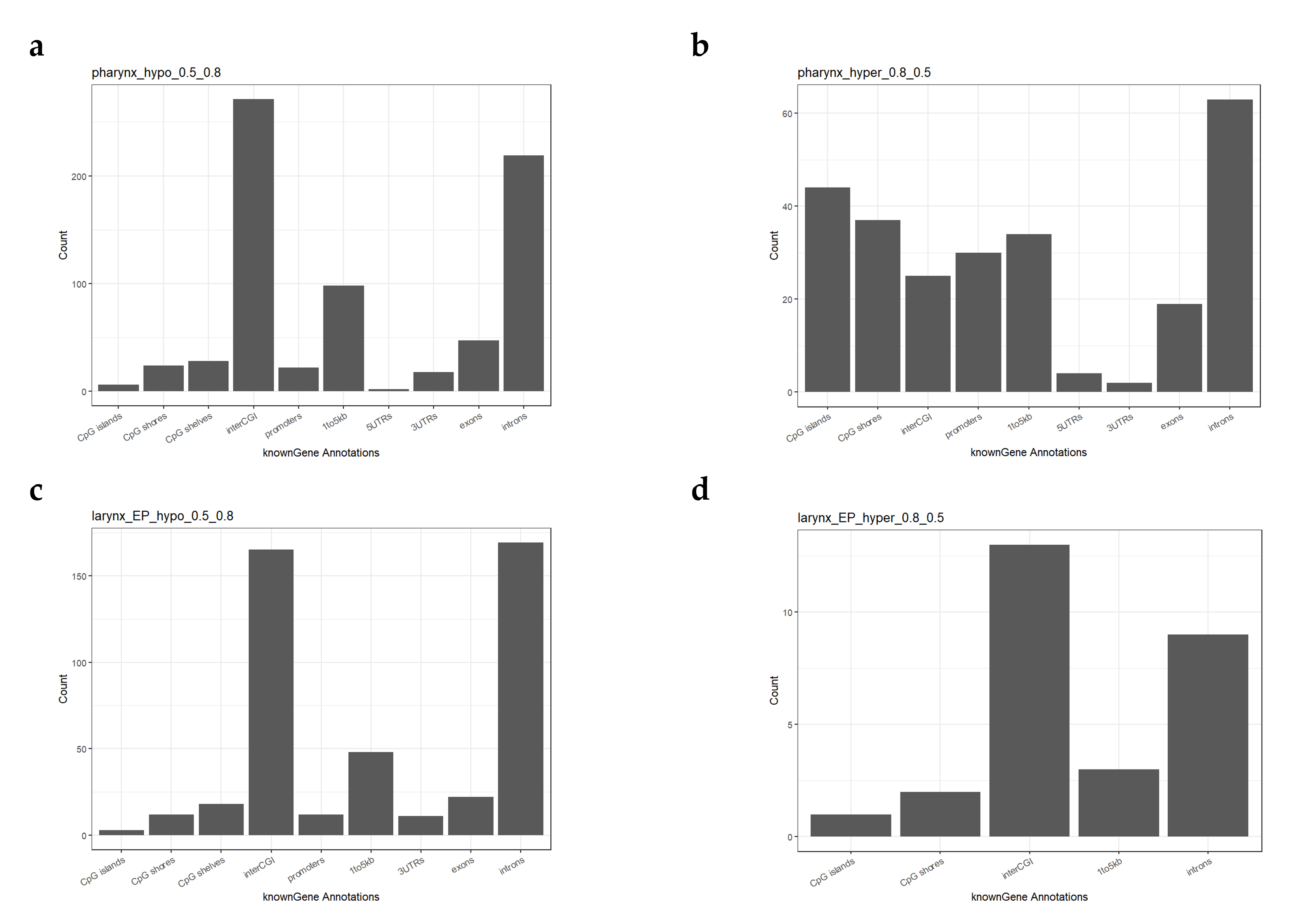

Supplement: Supplementary file 1 — Supplementary methods, figures and tables. [file jcav17p1318s1.zip › Supplementary Material files/Figures/FigureS2.tif]

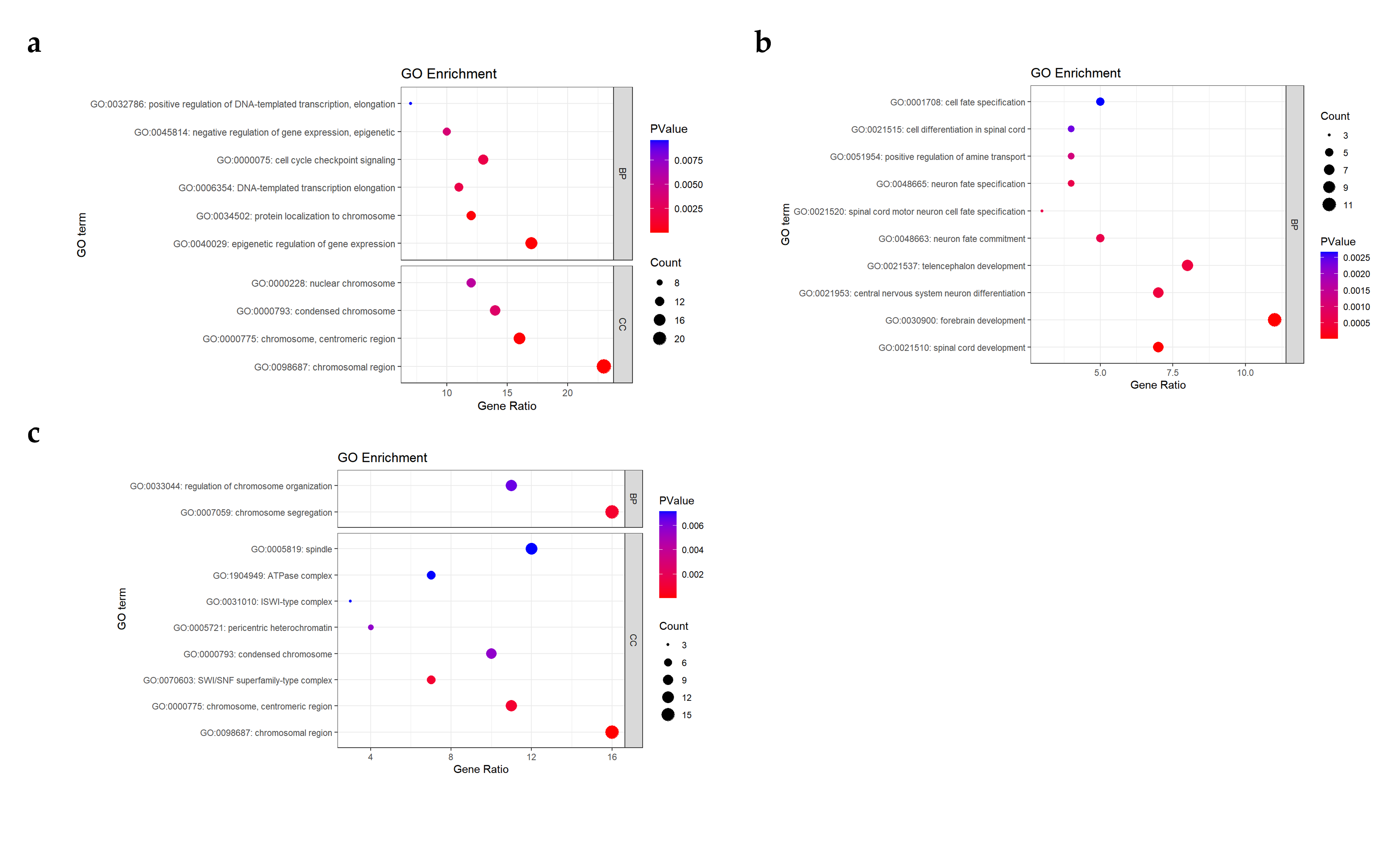

Supplement: Supplementary file 1 — Supplementary methods, figures and tables. [file jcav17p1318s1.zip › Supplementary Material files/Figures/FigureS3.tif]

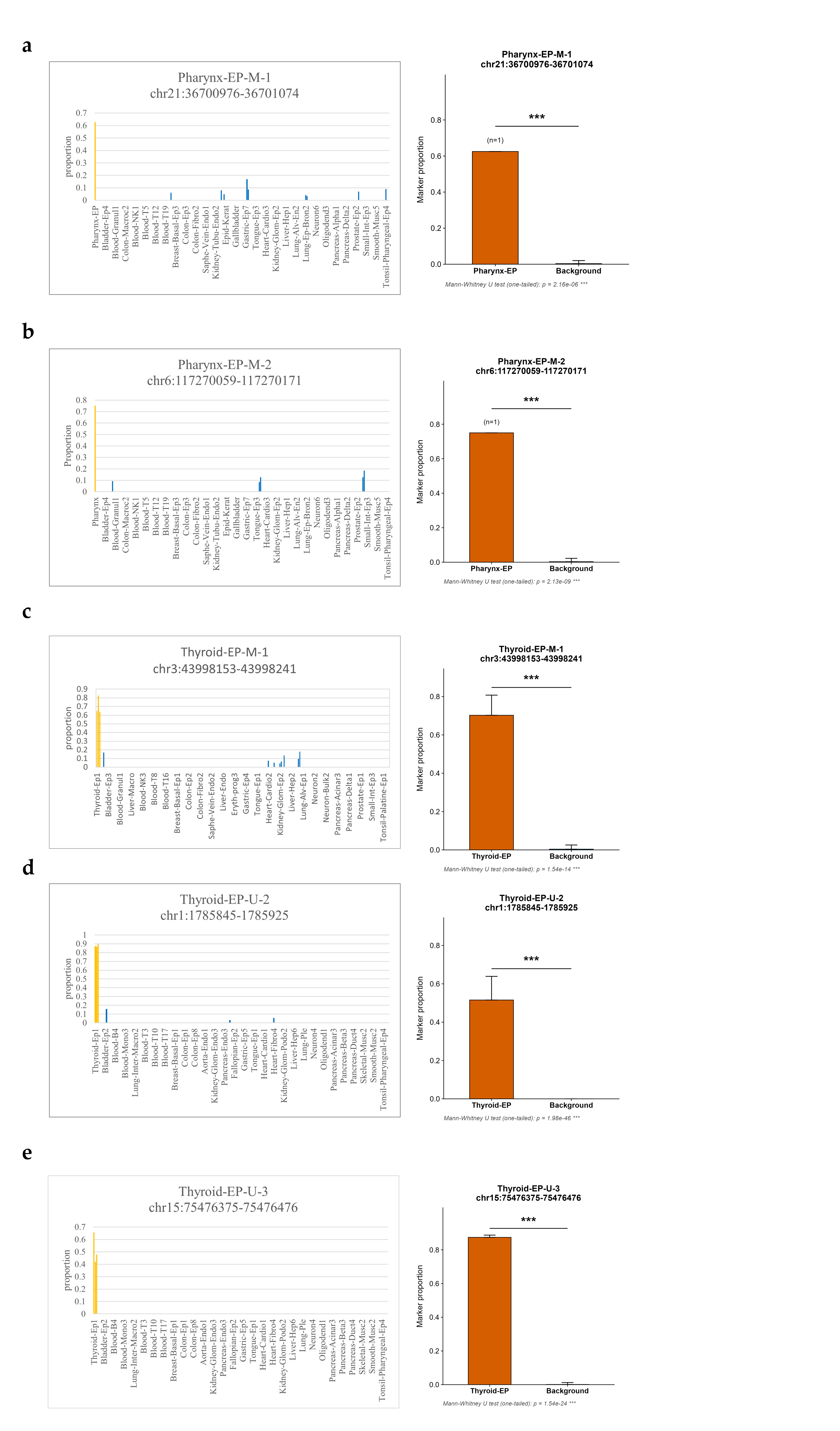

Supplement: Supplementary file 1 — Supplementary methods, figures and tables. [file jcav17p1318s1.zip › Supplementary Material files/Figures/FigureS4.tif]

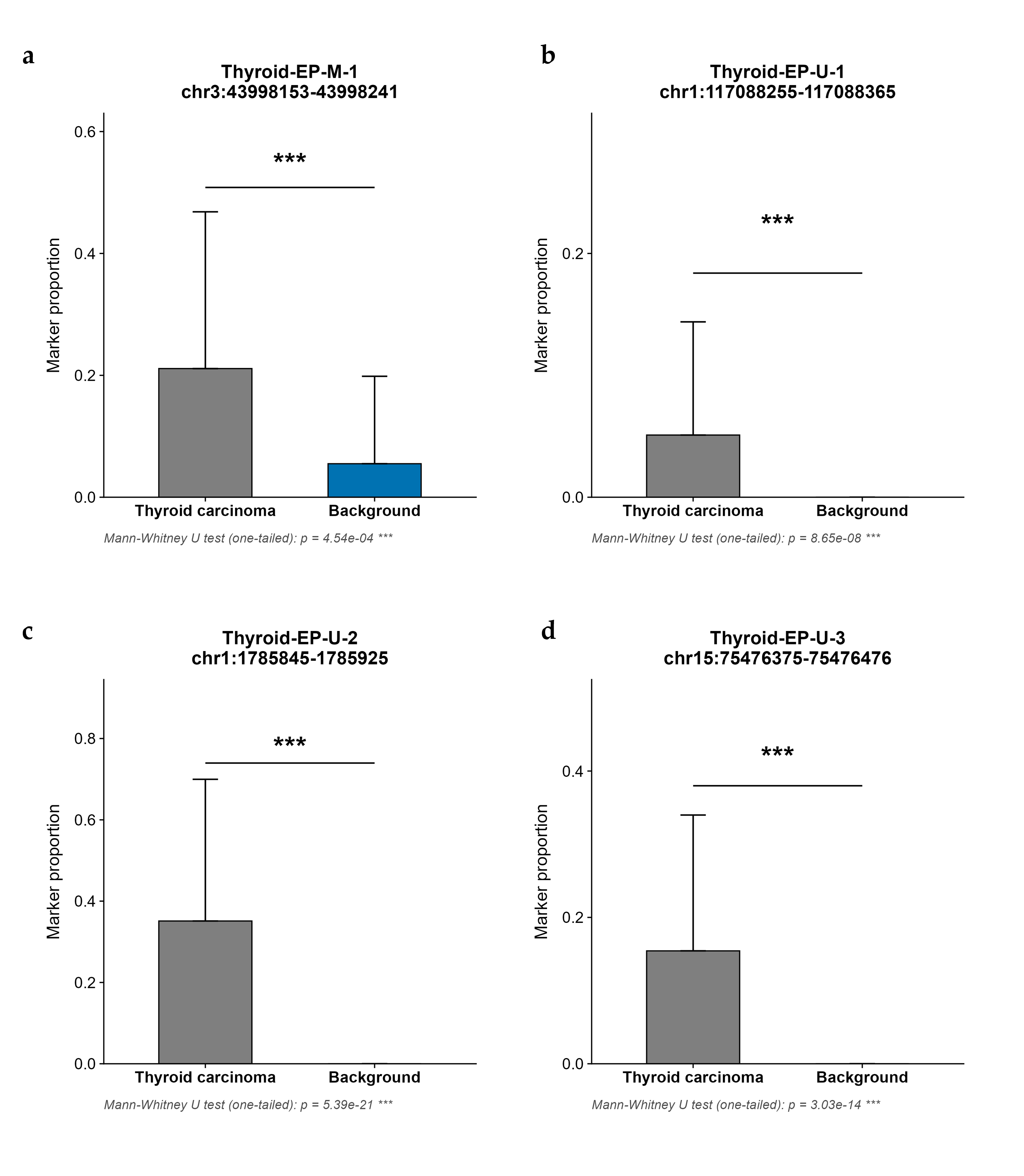

Supplement: Supplementary file 1 — Supplementary methods, figures and tables. [file jcav17p1318s1.zip › Supplementary Material files/Figures/FigureS5.tif]

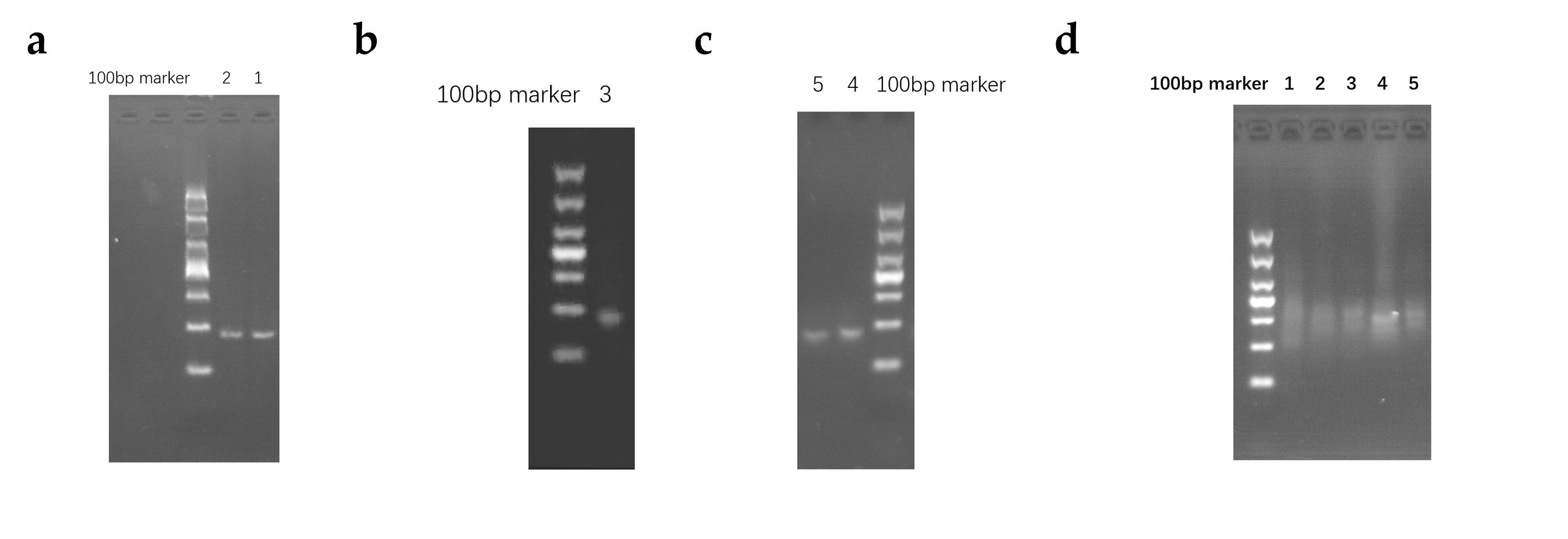

Supplement: Supplementary file 1 — Supplementary methods, figures and tables. [file jcav17p1318s1.zip › Supplementary Material files/Figures/FigureS6.tif]

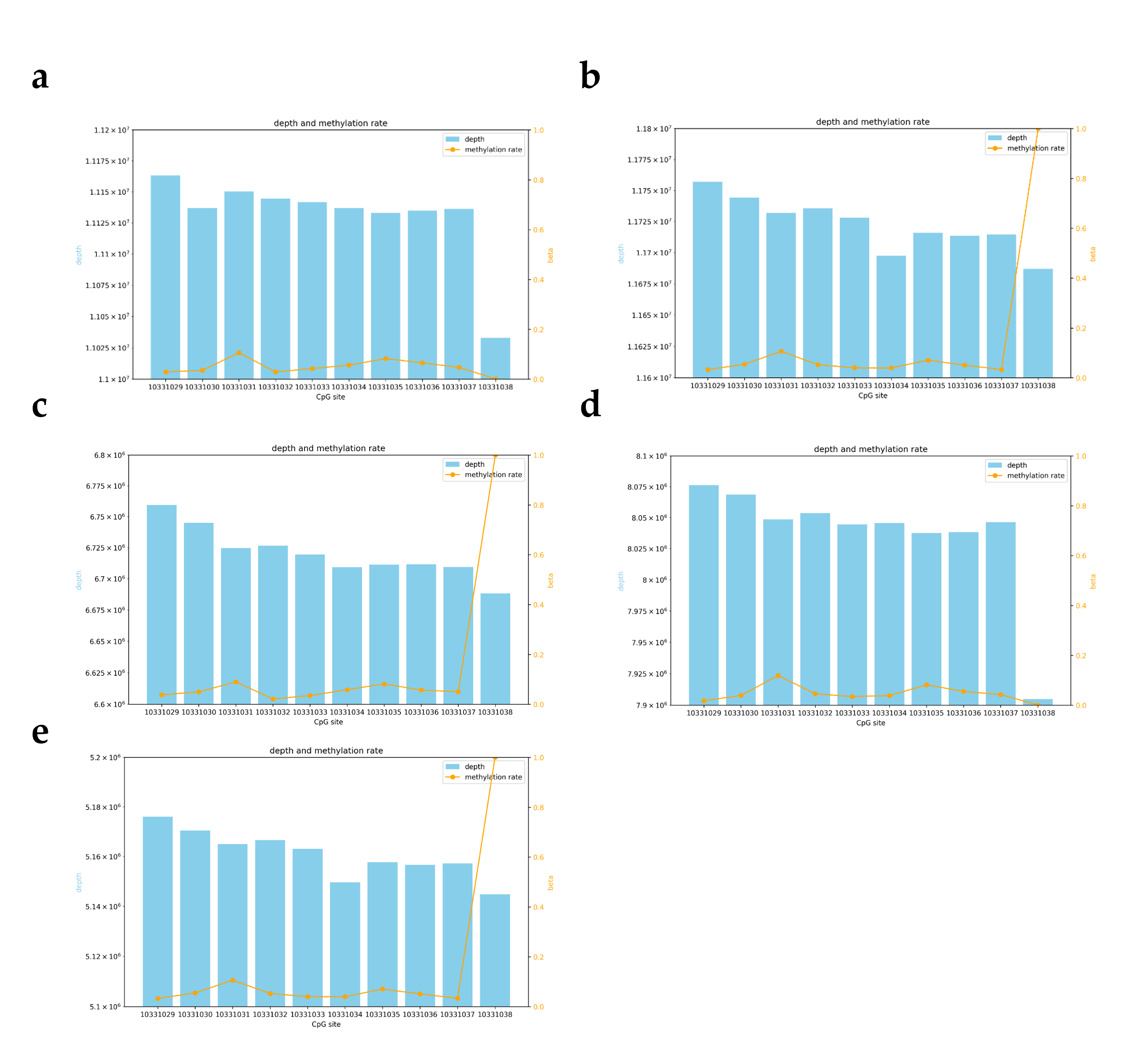

Supplement: Supplementary file 1 — Supplementary methods, figures and tables. [file jcav17p1318s1.zip › Supplementary Material files/Figures/FigureS7.tif]

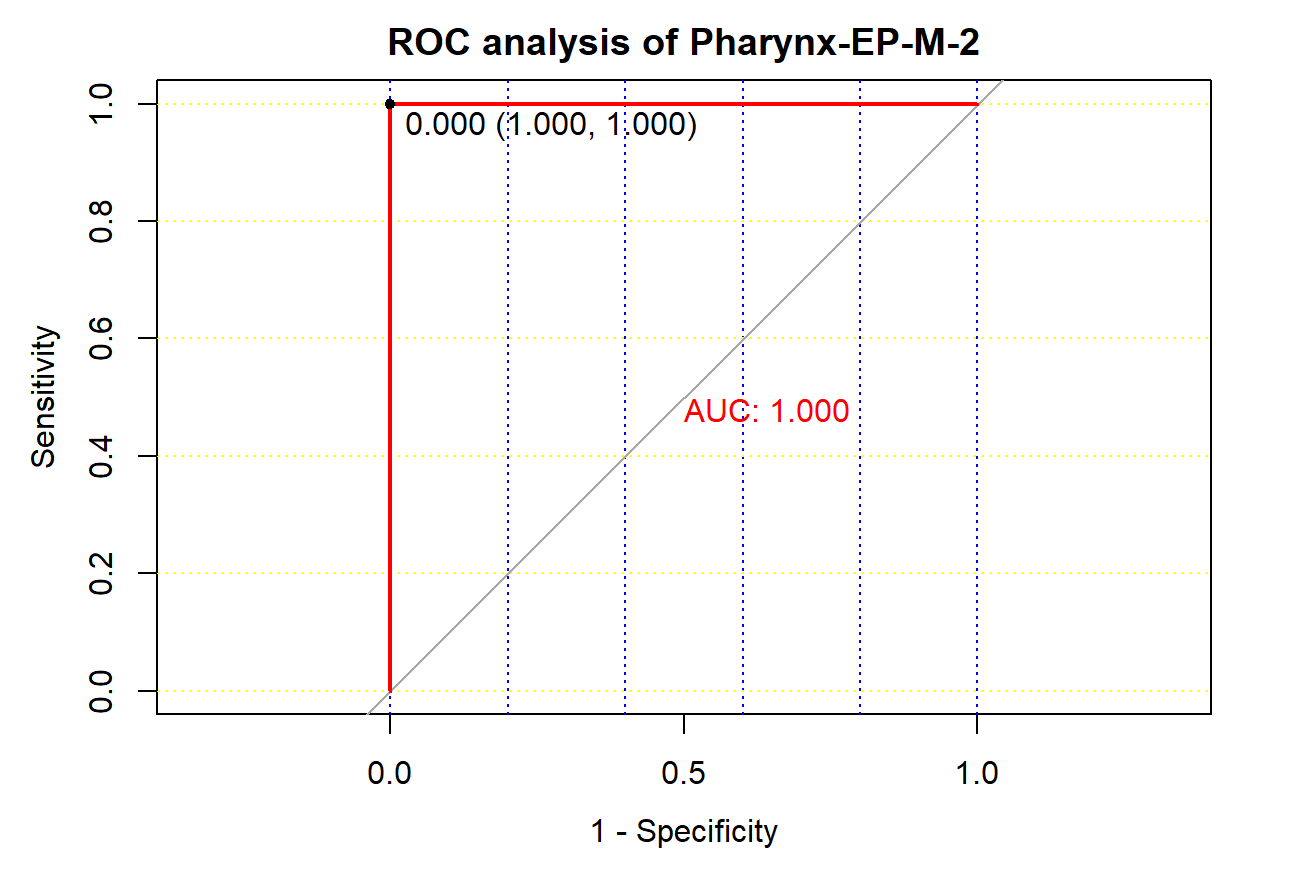

Supplement: Supplementary file 1 — Supplementary methods, figures and tables. [file jcav17p1318s1.zip › Supplementary Material files/Figures/FigureS8.tiff]
